# Investigating the association between low-density lipoprotein cholesterol/high-density lipoprotein cholesterol and the risk of carotid artery plaque in patients with first-ever ischemic stroke based on different glucose metabolic conditions

**DOI:** 10.3389/fendo.2026.1883414

**Published:** 2026-07-09

**Authors:** Lin Zhu, Min Wang, Xinrui Song, Tao Yan, Gaohan Zheng, Yue Tai, Ting Liu, Shencheng Luo, Bernhard Kolberg, Jing Li

**Affiliations:** 1 First Teaching Hospital of Tianjin University of Traditional Chinese Medicine, Tianjin, China; 2 National Clinical Research Center for Chinese Medicine, Tianjin, China; 3 Tianjin Medical University, Tianjin, China; 4 Tianjin Medical University General Hospital, Tianjin, China; 5 Department of Internal Medicine, Mannheim Medical School of Heidelberg University, Mannheim, Germany

**Keywords:** carotid plaque, exploratory mediation analysis, ischemic stroke, LDL-C/HDL-C, prediabetes

## Abstract

**Objective:**

This study investigated the association between the low-density lipoprotein cholesterol (LDL-C)/high-density lipoprotein cholesterol (HDL-C) ratio and carotid plaque risk in patients with first-ever ischemic stroke (IS). We focused on analyzing the differences in this association across different Glucose Metabolic Conditions, preliminarily explored the roles of glycated hemoglobin (HbA1c) and fasting plasma glucose (FPG) in this association, and thereby provided a basis for future prospective studies and risk assessment of IS events.

**Methods:**

This retrospective study included 12,166 patients hospitalized for first-time IS at the First Teaching Hospital of Tianjin University of Traditional Chinese Medicine from January 1, 2013, to May 1, 2023. The LDL-C/HDL-C–carotid plaque risk association across glucose metabolism statuses was assessed employing logistic regression, stratified analysis, and exploratory mediation analyses.

**Results:**

Of 12,166 participants, 9,469 (77.8%) had carotid plaque. Univariate logistic regression showed that LDL-C/HDL-C had a stronger association with the risk of carotid plaque formation than other lipid parameters (LDL-C, total cholesterol, and triglycerides). Multivariate analysis confirmed a significant positive association. Stratified analyses revealed that the association between LDL-C/HDL-C and plaque risk was relatively stronger in the Pre-DM cohort (odds ratio [OR]: 1.233, 95% confidence interval [CI]: 1.093–1.391), followed by patients with diabetes mellitus (OR: 1.178, 95% CI: 1.074–1.293); no significant association appeared in individuals with normal glucose regulation. Exploratory mediation analysis showed HbA1c and FPG accounted for 11.5% and 10.5% of the LDL-C/HDL-C–carotid plaque risk association, respectively.

**Conclusion:**

The findings of this study present that elevated LDL-C/HDL-C was significantly associated with carotid atherosclerosis in patients with IS, and this association was particularly stronger in the Pre-DM cohort. HbA1c and FPG had a certain moderating effect on this association, suggesting that the Pre-DM stage is a potential intervention window for focusing on LDL-C/HDL-C. LDL-C/HDL-C may serve as a reference indicator for clinically assessing the synergistic risk of “lipid-glucose co-morbidity,” which holds significant value for the secondary prevention of IS events.

## 1 Introduction

Atherosclerosis, a systemic chronic low-grade inflammatory disease with autoimmune features, underlies ischemic stroke (IS) pathology (1). It progresses through vascular endothelial dysfunction, lipid deposition, and inflammation, often starting in childhood and manifesting later in life (2). Multiple risk factors are known to drive atherosclerosis, with dyslipidemia and impaired glucose metabolism often coexisting, amplifying synergistic harm (3). Identifying modifiable risks and prevention strategies is thus critical for reducing atherosclerotic cardiovascular and cerebrovascular events and patient mortality.

Dyslipidemia, marked by altered blood lipids, drives atherosclerosis progression and is closely associated with IS. It typically features elevated total cholesterol (TC), low-density lipoprotein cholesterol (LDL-C), and triglycerides (TG), along with reduced high-density lipoprotein cholesterol (HDL-C) (4). While studies have emphasized single lipid markers (including TC, TG, LDL-C, and HDL-C), composite lipid parameters have shown greater predictive value recently. For example, the LDL-C/HDL-C ratio provides a comprehensive and dynamic reflection of atherosclerotic cerebrovascular disease risk compared to LDL-C or HDL-C alone (5). Elevated HDL-C is known to protect against carotid plaque and stroke events via reverse cholesterol transport function from peripheral tissues to the liver for metabolism or clearance (6). Furthermore, LDL-C has been strongly linked to carotid atherosclerosis (7). However, clinical observations have revealed substantial residual atherosclerotic risk even in patients with well-controlled LDL-C levels (8). The limited efficacy of individual lipid parameters for IS necessitates exploring the potential of the composite LDL-C/HDL-C ratio for atherosclerosis risk.

Insulin resistance (IR), a core pathophysiological driver of dysregulated glucose and lipid metabolism (9), raises glycated hemoglobin (HbA1c) and fasting plasma glucose (FPG) while promoting atherogenic dyslipidemia: low HDL levels, small dense LDL (sdLDL), elevated plasma TG levels, and postprandial hyperlipidemia (10). Glycemic markers such as HbA1c and FPG may thus play a certain role in the association between LDL-C/HDL-C and atherosclerotic risk. Prediabetes mellitus (Pre-DM) as a critical stage during which IR is active but diabetes mellitus (DM) has not developed, represents a potential window period for cerebrovascular disease intervention (11, 12). However, biomarker research in Pre-DM remains limited. Examining whether the association between LDL-C/HDL-C and carotid plaque is relatively more prominent in the Pre-DM population is thus essential for early identification of high-risk individuals.

Hence, this study aimed to investigate the LDL-C/HDL-C–carotid plaque risk association in patients with IS. Herein, the data from the First Teaching Hospital of Tianjin University of Traditional Chinese Medicine (12,166 patients with first-episode IS) were used. Additionally, we conducted an in-depth exploration of the association between LDL-C/HDL-C and carotid plaque risk under different glycemic control states (especially Pre-DM), and investigated the roles and respective contributions of HbA1c and FPG in this association. This study aimed to explore the potential value of LDL-C/HDL-C levels as an exploratory indicator of synergistic glucolipid metabolic risk, thereby providing a reference for the secondary prevention of IS events.

## 2 Research methods

### 2.1 Study population

**Figure 1:**
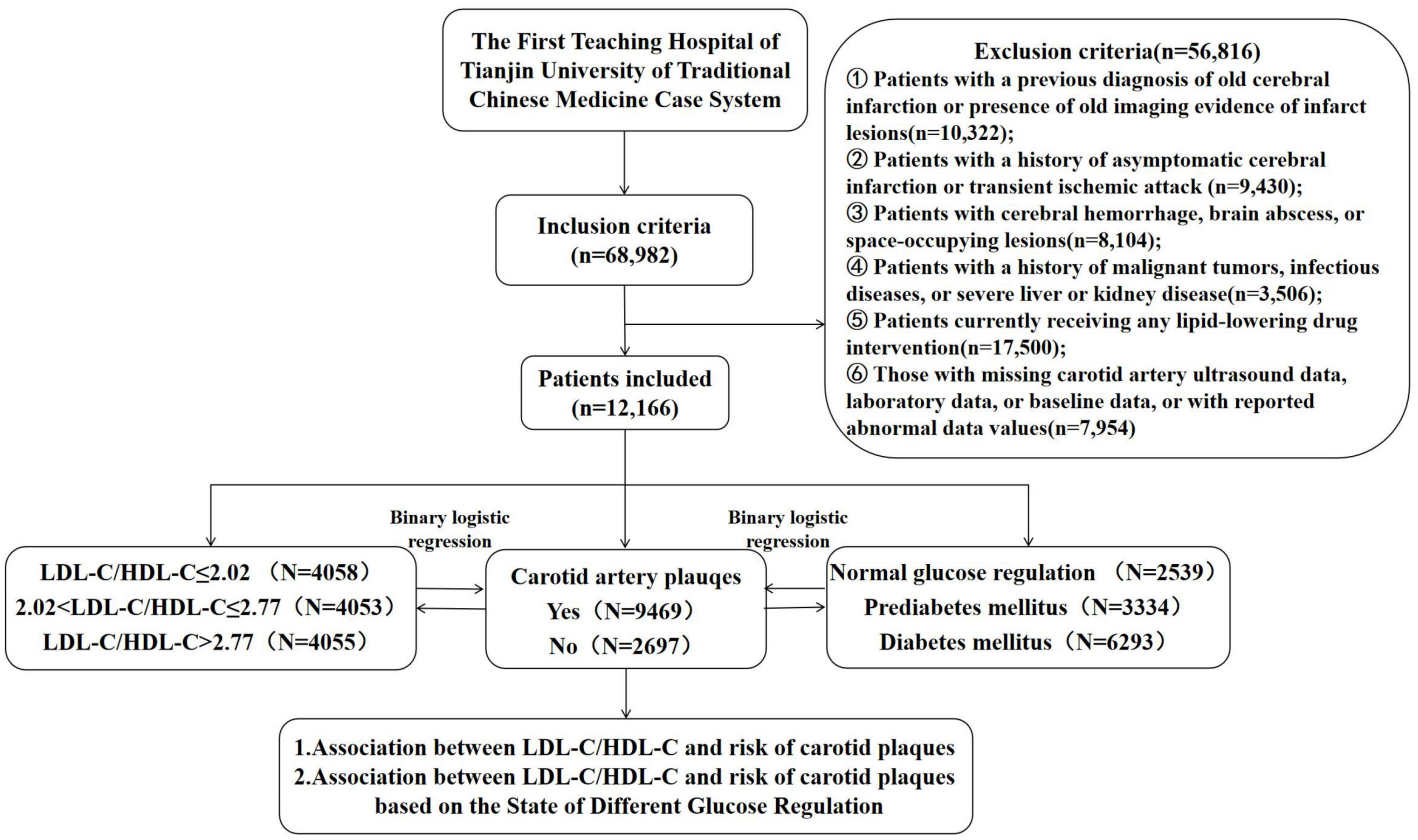
Flow chart depicting the enrollment process of the study participants.

This study conducted screening among 68,982 IS patients who were hospitalized at the First Teaching Hospital of Tianjin University of Traditional Chinese Medicine between January 1, 2013, and May 1, 2023. The screening process strictly adhered to the inclusion and exclusion criteria so as to ensure the study quality as well as to enhance the reliability of the results. Ultimately, this retrospective study enrolled 12,166 subjects. The screening flow is illustrated in **Figure 1**. This study was approved by the Ethics Committee of the First Teaching Hospital of Tianjin University of Traditional Chinese Medicine (Approval No.: TYLL2020[K]057) and registered with the China Clinical Trial Registry (Registration No.: ChiCTR2100045415).

### 2.2 Standards

#### 2.2.1 Inclusion criteria

①Meets the 2018 Chinese Guidelines for Diagnosis and Treatment of Acute Ischemic Stroke with respect to IS diagnostic criteria and magnetic resonance imaging (MRI) results (13). ②Every case record is fully documented and can be tracked.③Lipids levels must meet any one of the following corresponding criteria (to exclude the influence of lipid-lowering drug intervention on the natural progression of lipid profiles):

- Newly diagnosed individuals not on medication: This group includes individuals who have recently been diagnosed with dyslipidemia and have not yet started any statins or other lipid-lowering drug therapy.
- Individuals with a history of diagnosis but no intervention: This category consists of individuals who have been diagnosed with dyslipidemia but have not received any lipid-lowering medication treatment in the past six months.
- Individuals with normal indicators and no prior interventions: This group is characterized by individuals whose lipids levels are within normal ranges, and who have never undergone any lipid-lowering interventions.

#### 2.2.2 Exclusion criteria

① Patients with a previous diagnosis of old cerebral infarction or presence of old imaging evidence of infarct lesions;
② Patients with a history of asymptomatic cerebral infarction or transient ischemic attack;
③ Patients with cerebral hemorrhage, brain abscess, or space-occupying lesions;
④ Patients with a history of malignant tumors, infectious diseases, or severe liver or kidney disease;
⑤ Patients currently receiving any lipid-lowering drug intervention;
⑥ Those with missing carotid artery ultrasound data, laboratory data, or baseline data, or with reported abnormal data values.

### 2.3 Assessment of atherosclerosis

A professional sonographer, blinded to the subject’s specific clinical information, employed B-mode ultrasound to scan the common carotid artery, internal carotid artery, and carotid bifurcation in order to evaluate the extent of carotid atherosclerosis. Intima-media thickness (IMT) was measured as the vertical distance from the intima–lumen echo interface to the media–adventitia echo interface. Carotid IMT specifically refers to the average IMT value of both sides of the common carotid artery. In accordance with the Mannheim Consensus, carotid plaque was defined by two criteria: either the presence of focal wall thickening ≥0.5 mm or ≥50% thickening relative to the surrounding vessel wall; or IMT ≥1.5 mm above the luminal–intimal or media–intimal interface distance (14). Based on color Doppler ultrasound imaging outcomes, plaques were categorized by quantity [single (n = 1) or multiple (n ≥ 2)] and echogenicity (hypoechoic, hyperechoic, or mixed echo).

### 2.4 Potential covariates

The baseline data were collected from the study subjects by trained medical professionals via structured questionnaires. (1) Demographic and lifestyle data: Including age, sex, smoking history, and alcohol consumption history. (2) Medical conditions and medication history: Past medical conditions such as hypertension and diabetes, along with information on antihypertensive and antidiabetic medications. (3) Physical examination and specialist assessments: Systolic blood pressure (SBP), diastolic blood pressure (DBP), and National Institutes of Health Stroke Scale (NIHSS). (4) Laboratory data: Including the levels of HbA1c, FPG, TC, TG, HDL-C, and LDL-C.

Blood pressure measurements were performed by experienced physicians using automated sphygmomanometers. Hypertension was diagnosed as SBP ≥130 mmHg or DBP ≥80 mmHg (15). Diabetes was diagnosed if any one criterion was met: (1) FPG ≥7 mmol/L or (2) HbA1c ≥6.5% or (3) self-reported DM diagnosed by a physician. Pre-DM was characterized by 5.56 ≤ FPG < 7 mmol/L or HbA1c 5.7–6.4%. Normal glucose regulation (NGR) was defined as FPG <5.56 mmol/L and HbA1c <5.7% (16). At the time of admission, patient severity was assessed with reference to the NIHSS score (0–24 points), where higher scores indicated more severe stroke (17). Laboratory data were directly measured by medical staff using automated blood analyzers. The exposure variable LDL-C/HDL-C was calculated from the traditional lipid parameters LDL-C and HDL-C, thereby comprehensively reflecting the dynamic balance between anti-atherogenic and pro-atherogenic lipoproteins.

### 2.5 Quality control

Data collection strictly adhered to the inclusion and exclusion criteria. An intelligent data management platform systematically screened the eligible participant data. Ultrasound examinations were performed and reported by the same professional technician with over five years of work experience. The equipment, parameters, and diagnostic criteria remained consistent throughout the study period. Daily equipment calibration and maintenance were handled by dedicated personnel, and regular quality control assessments were conducted to ensure the consistency and reliability of the measurements. Two professionals jointly reviewed and cross-verified all medical records to ensure data authenticity and validity. This study employed complete case analysis, including only patients with complete key variables. Missing data primarily originated from laboratory indicators, ultrasound measurements, and key covariates, and were considered missing at random (MAR). No significant differences were observed in major baseline characteristics between the included and excluded populations, suggesting that the risk of selection bias introduced by complete case analysis was manageable. In addition, a dual-verification mechanism was implemented for data entry, wherein two researchers independently evaluated both the raw data and subsequent value assignments to guarantee accuracy.

### 2.6 Statistical analysis

The baseline characteristics of the participants were analyzed descriptively after stratification by LDL-C/HDL-C tertiles. Specifically, for intergroup comparisons, categorical variables were assessed using chi-square (χ²) tests, whereas continuous variables with skewed distributions were analyzed through nonparametric rank-sum tests (Kruskal–Wallis H). Multivariate logistic regression models assessed the association between the LDL-C/HDL-C levels and carotid plaque risk, yielding the corresponding odds ratios (ORs) and 95% confidence intervals (CIs). In addition, the P-trend values were calculated by incorporating the midpoint of each LDL-C/HDL-C tertile as a continuous variable into the model. Two multivariate logistic regression models were constructed to facilitate comprehensive analysis: Model 1 (unadjusted) and Model 2 (adjusted for sex, age, smoking, alcohol consumption, hypertension, NIHSS score, TC, and TG). The predictive performance of the LDL-C/HDL-C ratio for the risk of carotid artery plaque was evaluated using the receiver operating characteristic (ROC) curve. Its incremental predictive value was further assessed using the net reclassification improvement (NRI) and integrated discrimination improvement (IDI) indices.

We evaluated the strength of the relationship between LDL-C/HDL-C and carotid plaque formation across different glycemic statuses (NGR, Pre-DM, and DM). Restricted cubic spline (RCS) analysis was used to test the linear assumption and clarify the dose-response relationship between them. Furthermore, exploratory mediation analysis was performed to preliminarily investigate whether HbA1c and FPG play a certain role in the association between LDL-C/HDL-C and carotid plaque risk. Finally, subgroup analysis was used to verify the consistency of this association across different populations. In the sensitivity analysis, a Poisson regression model with robust error variance was used to calculate the prevalence ratio (PR), and E-value analysis was performed to assess the robustness of the study conclusions.

All statistical analyses were performed using R software (version 3.4.3) and SPSS 27.0 (IBM Corp, Armonk, NY, USA). P < 0.05 was considered to indicate statistical significance.

## 3 Results

### 3.1 Baseline characteristics

This study included 12,166 patients with first-time IS. Of these, 8,306 (68.2%) were aged ≥60 years, 7,805 (64.2%) were male, and 77.8% had carotid plaque. We stratified participants into three groups based on LDL-C/HDL-C tertiles: T1 (LDL-C/HDL-C ≤ 2.02), T2 (2.02 < LDL-C/HDL-C ≤ 2.77), and T3 (LDL-C/HDL-C > 2.77). Compared with the T1 group, the T3 group had higher proportions of males, smokers, and drinkers, as well as greater rates of past diabetes history and antidiabetic medication use (all P < 0.05; **Table 1**). Patients in the T3 group also showed significantly higher levels of SBP, DBP, HbA1c, FPG, TC, TG, and LDL-C, but lower HDL-C levels (all P < 0.001). These findings suggest a comorbidity trend linking elevated LDL-C/HDL-C ratios with impaired glucose metabolism.

**Table 1:** General characteristics of the research participants.

Characteristics	Total (N = 12,166)	LDL-C/HDL-C			P-value
		T1 (N = 4058)	T2 (N = 4053)	T3 (N = 4055)	
Sex, n (%)					<0.001
Male	7805 (64.2)	2474 (61.0)	2613 (64.5)	2718 (67.0)	
Female	4361 (35.8)	1584 (39.0)	1440 (35.5)	1337 (33.0)	
Age, years	64.0 (58.0–72.0)	65.0 (58.0–73.0)	64.0 (58.0–72.0)	64.0 (57.0–72.0)	<0.001
<60, n (%)	8306 (68.3)	2921 (72.0)	2756 (68.0)	2629 (64.8)	
≥60, n (%)	3860 (31.7)	1137 (28.0)	1297 (32.0)	1426 (35.2)	
DBP, mmHg	86.0 (79.0–94.0)	86.0 (78.0–94.0)	87.0 (79.0–94.0)	87.0 (79.0–95.0)	<0.001
SBP, mmHg	147.0 (134.0–161.0)	146.0 (132.0–161.0)	147.0 (134.0–161.0)	148.0 (135.0–163.0)	<0.001
NIHSS	4.0 (2.0–7.0)	5.0 (2.0–8.0)	5.0 (2.0–7.0)	4.0 (2.0–7.0)	0.005
HbA1c, %	6.2 (5.6–7.4)	6.1 (5.6–7.2)	6.2 (5.6–7.3)	6.4 (5.7–7.8)	<0.001
FPG, mmol/L	5.7 (4.9–7.3)	5.5 (4.8–6.9)	5.6 (4.9–7.2)	6.0 (5.0–7.9)	<0.001
TC, mmol/L	4.1 (3.4–4.9)	3.4 (2.9–4.1)	4.1 (3.5–4.8)	4.8 (4.2–5.6)	<0.001
TG, mmol/L	1.3 (1.0–1.8)	1.1 (0.8–1.4)	1.3 (1.0–1.8)	1.6 (1.2–2.1)	<0.001
HDL-C, mmol/L	1.0 (0.9–1.2)	1.1 (0.9–1.4)	1.0 (0.9–1.2)	0.9 (0.8–1.1)	<0.001
LDL-C, mmol/L	2.4 (1.8–3.1)	1.7 (1.4–2.1)	2.4 (2.0–2.9)	3.2 (2.7–3.7)	<0.001
Smoking, n (%)	5967 (49.0)	1830 (45.1)	1972 (48.7)	2165 (53.4)	<0.001
Drinking, n (%)	4850 (39.9)	1523 (37.5)	1615 (39.8)	1712 (42.2)	<0.001
History of hypertension, n (%)	8151 (67.0)	2739 (67.5)	2724 (67.2)	2688 (66.3)	0.482
History of diabetes, n (%)	4965 (40.8)	1636 (40.3)	1605 (39.6)	1724 (42.5)	0.021
Use of antihypertensive medication, n (%)	8295 (68.2)	2783 (68.8)	2773 (68.4)	2739 (67.5)	0.561
Use of antidiabetic medication, n (%)	5008 (41.2)	1646 (40.6)	1624 (40.1)	1738 (42.9)	0.024
Carotid Plaque, n (%)	9469 (77.8)	3100 (76.4)	3122 (77.0)	3247 (80.1)	<0.001
Number of Carotid Plaques, n (%)					<0.001
0	2697 (22.2)	958 (23.6)	931 (23.0)	808 (19.9)	
1	1863 (15.3)	664 (16.4)	634 (15.6)	565 (13.9)	
≥2	7606 (62.5)	2436 (60.0)	2488 (61.4)	2682 (66.1)	
Hypoechoic plaque	1844 (15.2)	555 (13.7)	612 (15.1)	677 (16.7)	<0.001
Hyperechoic plaque	2483 (20.4))	905 (22.3)	798 (19.7)	780 (19.2)	0.001
Mixture plaque	3436 (28.2)	1258 (31.0)	1140 (28.1)	1038 (25.6)	<0.001

T1: LDL-C/HDL-C ≤ 2.02, T2: 2.02 < LDL-C/HDL-C ≤ 2.77, T3: LDL-C/HDL-C > 2.77.

DBP, diastolic blood pressure; SBP, systolic blood pressure; NIHSS, National Institutes of Health Stroke Scale; HbA1c, glycosylated hemoglobin; FPG, fasting plasma glucose; TC, total cholesterol; TG, triglycerides; HDL-C, high-density lipoprotein cholesterol; LDL-C, low-density lipoprotein cholesterol.

The comparison of baseline characteristics between the included and excluded populations showed no significant differences between the two groups in terms of sex, age, smoking history, drinking history, past medical history, lipid parameters, glycemic parameters, or plaque prevalence (P > 0.05), suggesting that the impact of selection bias on the study conclusions was limited (**Supplementary Table 1**).

### 3.2 Univariate analysis of risk factors for carotid plaque

Univariate logistic regression showed a significant association of sex, age, smoking, drinking status, previous diagnoses of hypertension and diabetes, and use of antihypertensive and antidiabetic medications with increased carotid plaque risk (P < 0.001). Among lipid parameters, the LDL-C/HDL-C ratio showed a significant positive correlation with plaque risk (OR: 1.154, 95% CI: 1.099–1.212, P < 0.001), with a larger effect size than individual lipids or other non-traditional markers. This supports its use as the primary exposure variable in subsequent analyses (**Table 2**).

**Table 2:** Risk analysis carotid artery plaques.

Characteristic	Risk of carotid plaques	
	OR (95%CI)	P-value
Age	1.075 (1.070–1.080)	<0.001
<60	Reference	
≥60	3.230 (2.956–3.529)	
Sex		<0.001
Female	Reference	
Male	1.559 (1.429–1.701)	
Smoking	1.482 (1.359–1.616)	<0.001
Drinking	1.307 (1.195–1.428)	<0.001
History of hypertension	1.390 (1.272–1.519)	<0.001
History of diabetes	1.474 (1.348–1.612)	<0.001
Use of antihypertensive medication	1.403 (1.283–1.534)	<0.001
Use of antidiabetic medication	1.467 (1.342–1.605)	<0.001
SBP	1.005 (1.003–1.007)	<0.001
DBP	0.982 (0.978–0.985)	<0.001
NIHSS	1.055 (1.043–1.068)	<0.001
FPG	1.055 (1.035–1.075)	<0.001
HbA1c	1.095 (1.063–1.128)	<0.001
TC	0.985 (0.950–1.022)	0.427
TG	0.929 (0.898–0.961)	<0.001
LDL-C	1.036 (0.988–1.086)	0.149
HDL-C	0.625 (0.539–0.726)	<0.001
TC/HDL-C	1.094 (1.053–1.136)	<0.001
TG/HDL-C	0.964 (0.939–0.990)	0.008
LDL-C/HDL-C	1.154 (1.099–1.212)	<0.001

DBP, diastolic blood pressure; SBP, systolic blood pressure; NIHSS, National Institutes of Health Stroke Scale; HbA1c, glycosylated hemoglobin; FPG, fasting plasma glucose; TC, total cholesterol; TG, triglycerides; HDL-C, high-density lipoprotein cholesterol; LDL-C, low-density lipoprotein cholesterol.

### 3.3 Relationship between LDL-C/HDL-C and carotid plaque risk

Both before and after adjustment, continuous LDL-C/HDL-C showed a significant positive association with carotid plaque risk. As a categorical variable, the T3 group had a 1.242-fold (unadjusted) and 1.296-fold (adjusted) higher risk than the T1 group. Trend analysis confirmed consistent associations for LDL-C/HDL-C (continuous and categorical) with carotid plaque occurrence, before and after confounder adjustment (P for trend <0.001) (**Table 3**).

**Table 3:** Relationship between LDL-C/HDL-C and the risk of carotid plaques.

Characteristic	Risk of carotid plaques			
	OR (95% CI)^a^	P-value	OR (95% CI)^b^	P-value
LDL-C/HDL-C	1.154 (1.099–1.212)	<0.001	1.224 (1.147–1.305)	<0.001
T1	Reference		Reference	
T2	1.036 (0.935–1.149)	0.497	1.061 (0.945–1.191)	0.319
T3	1.242 (1.117–1.380)	<0.001	1.296 (1.134–1.481)	<0.001
P for trend		<0.001		<0.001

T1: LDL-C/HDL-C ≤ 2.02, T2: 2.02 < LDL-C/HDL-C ≤ 2.77, T3: LDL-C/HDL-C > 2.77.

OR, Odds ratio; 95%CI, 95% Confidence interval.

model a: unadjusted for any covariates;.

model b: adjusted for sex, age, smoking status, drinking status, hypertension, NIHSS, TC, TG.

### 3.4 Incremental predictive value of LDL-C/HDL-C for carotid artery plaque

To evaluate the incremental predictive value of the LDL-C/HDL-C ratio for carotid artery plaque beyond traditional risk factors, this study constructed a receiver operating characteristic (ROC) curve. The model including only traditional clinical risk factors (sex, age, smoking status, drinking status, hypertension, NIHSS, TC, TG) yielded an area under the ROC curve (AUC) of 0.702 (95% CI: 0.691–0.714). After adding LDL-C/HDL-C to the traditional factors, the model AUC increased to 0.705 (95% CI: 0.694–0.717). The likelihood ratio test showed a significant improvement in model fit (χ² = 30.8, df = 1, P < 0.001). Combined with the net reclassification improvement (NRI: 0.015, 95% CI: 0.003–0.028, P = 0.007) and the integrated discrimination improvement (IDI: 0.0026, 95%CI: 0.0016-0.0037, P < 0.001), these results suggest that LDL-C/HDL-C provides certain incremental predictive value for identifying the risk of carotid artery plaque beyond traditional factors (**Table 4**; **Supplementary Table 2**).

**Table 4:** Incremental predictive value of LDL/C-HDL-C for carotid plaque risk: comparison of ROC curve parameters.

Model	AUC (95% CI)	P-value	Optimal cutoff value	Youden index	Specificity	Sensitivity
Clinical Risk Factors	0.702 (0.691-0.714)	Reference	0.771	0.303	0.621	Reference
Clinical Risk Factors +LCL-C/HDL-C	0.705 (0.694-0.717)	0.0019	0.779	0.307	0.648	0.659

model: adjusted for sex, age, smoking status, drinking status, hypertension, NIHSS, TC, TG.

### 3.5 Stratified analysis across different glycemic statuses

Among the 12,166 participants, 2,539 (20.8%) had NGR, 3,334 (27.4%) had Pre-DM, and 6,293 (51.7%) had DM. After confounder adjustment, the association between LDL-C/HDL-C and carotid plaque risk was stronger in the Pre-DM group (OR: 1.233, 95% CI: 1.093–1.391), followed by that of DM (OR: 1.178, 95% CI: 1.074–1.293); no significant association appeared in patients with NGR. As a categorical variable, only patients with Pre-DM showed a significant association between the LDL-C/HDL-C ratios. Specifically, the plaque formation risk in the T3 group was 1.384 times that of T1 (OR: 1.384, 95% CI: 1.081–1.771, P = 0.010) (**Table 5**).

**Table 5:** Association between LDL-C/HDL-C and the risk of carotid plaques according to different glucose regulation states.

Glucose regulation state	Characteristics	Risk of carotid plaques			
		OR(95%CI)^a^	P-value	OR(95%CI)^b^	P-value
NGR	LDL-C/HDL-C	1.070(0.963–1.188)	0.208	1.125(0.977–1.296)	0.102
	T1	Reference		Reference	
	T2	0.981(0.800–1.201)	0.850	1.013(0.800–1.284)	0.912
	T3	1.075(0.863–1.338)	0.520	1.174(0.887–1.555)	0.263
	P for trend		0.558		0.286
Pre-DM	LDL-C/HDL-C	1.161(1.059–1.273)	0.001	1.233(1.093–1.391)	<0.001
	T1	Reference		Reference	
	T2	1.200(0.993–1.451)	0.059	1.277(1.032–1.581)	0.025
	T3	1.294(1.064–1.574)	0.010	1.384(1.081–1.771)	0.010
	P for trend		0.009		0.009
DM	LDL-C/HDL-C	1.148(1.071–1.231)	<0.001	1.178(1.074–1.293)	<0.001
	T1	Reference		Reference	
	T2	0.950(0.814–1.110)	0.521	0.925(0.777–1.100)	0.376
	T3	1.194(1.021–1.395)	0.026	1.129(0.926–1.375)	0.230
	P for trend		0.022		0.219

T1: LDL-C/HDL-C ≤ 2.02, T2: 2.02 < LDL-C/HDL-C ≤ 2.77, T3: LDL-C/HDL-C > 2.77.

OR, Odds ratio; 95%CI, 95% Confidence interval.

model a: unadjusted for any covariates; model b: adjusted for sex, age, smoking status, drinking status, hypertension, NIHSS, TC, TG.

RCS curves revealed significant linear dose–response relationships between LDL-C/HDL-C and plaque risk in both Pre-DM and DM populations (P for nonlinearity >0.05), indicating that plaque risk continuously increases with the elevation of the LDL-C/HDL-C ratio; no significant association was observed in the NGR population (**Supplementary Figure S1**).

### 3.6 Exploratory mediation analysis of glycemic markers HbA1c and FPG

**Figure 2:**
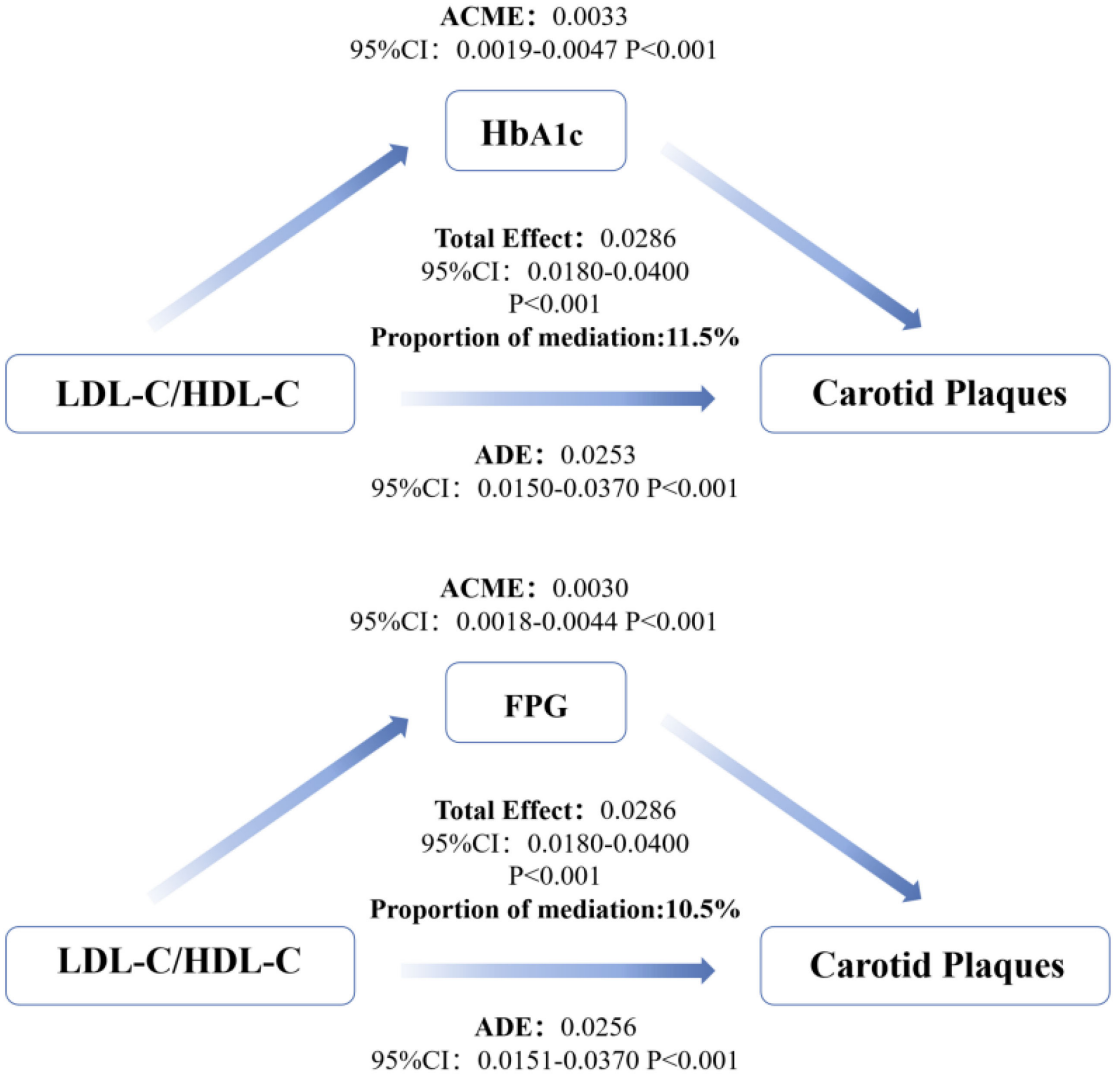
The Exploratory Mediation Analysis of HbA1c and FPG on the Relationship Between LDL-C/HDL-C and the Risk of Carotid Plaques. This analysis is exploratory based on cross-sectional data; causal inference is not implied.

To evaluate whether HbA1c and FPG play a partial role in the association between the LDL-C/HDL-C ratio and the risk of carotid artery plaque, this study conducted an exploratory mediation analysis using the Bootstrap method (1,000 resampling iterations). LDL-C/HDL-C showed a significant positive correlation to carotid plaque formation (Total Effect: 0.0286, 95% CI: 0.0180–0.0400, P < 0.001). Meanwhile, LDL-C/HDL-C indirectly increased the risk of plaque formation through elevated HbA1c and FPG, with effect proportions of 11.5% and 10.5%, respectively (P < 0.001). Notably, after controlling for the effects of HbA1c and FPG, the influence of LDL-C/HDL-C on carotid plaque risk remained significant (**Figure 2**; **Supplementary Table 3**).

### 3.7 Subgroup analysis

**Figure 3:**
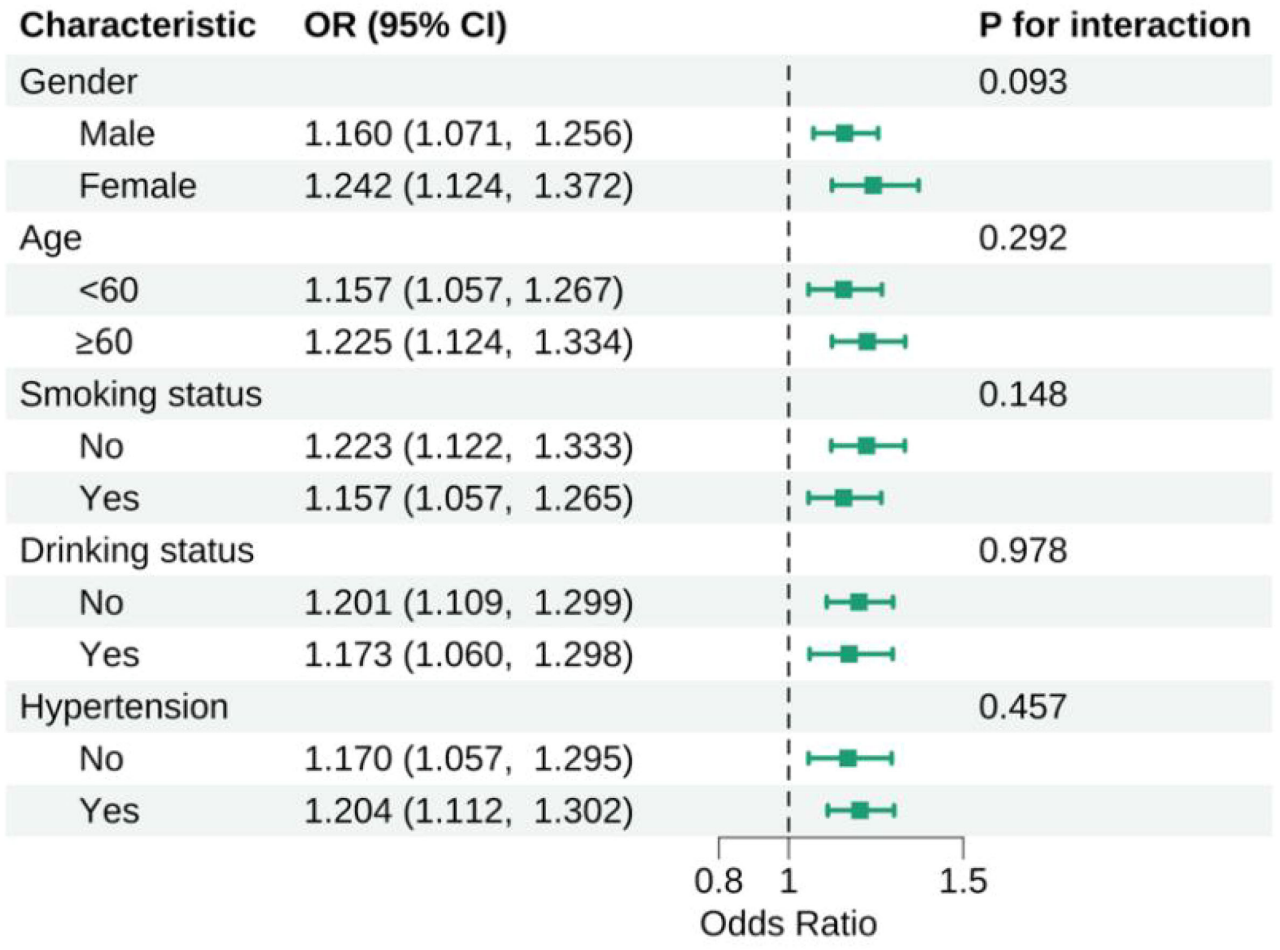
Subgroup analysis of the association between LDL-C/HDL-C and the risk of Carotid Plaques. OR, Odds ratio; 95%CI, 95% Confidence interval. Adjusted for sex, age, smoking status, drinking status, hypertension, NIHSS, TC, TG.

To test the robustness of the LDL-C/HDL-C–carotid plaque risk association, we performed subgroup analyses stratified by sex, age, smoking status, drinking status, and prior hypertension history. Interestingly, plaque risk increased consistently with higher LDL-C/HDL-C across all subgroups (all P < 0.05). No significant interactions were observed across all subgroups (all P for interaction > 0.05), indicating consistent associations regardless of patient characteristics. No meaningful differences in effect sizes were observed, which supports the robustness of the study findings (**Figure 3**).

### 3.8 Sensitivity analysis

To verify the reliability of the study findings, sensitivity analyses were performed. First, a Poisson regression model with robust error variance was used to assess the robustness of the study conclusions given the high prevalence. After adjusting for covariates, the prevalence ratio (PR) was 1.033 (95% CI: 1.021–1.045, P < 0.001), which was consistent in direction with the main analysis and statistically significant (**Supplementary Table 4**). Second, an E-value analysis was conducted to quantitatively assess the potential impact of unmeasured confounding factors on the robustness of the results. The results showed that the E-value for the point estimate was 1.26, and the E-value for the lower bound of the 95% confidence interval was 1.21, suggesting that future studies could collect variables such as statin use and BMI to further improve confounding control (**Supplementary Table 5**).

## 4 Discussion

This large-scale retrospective study demonstrated that in patients with first-episode IS, the LDL-C/HDL-C ratio was significantly positively associated with carotid plaque risk, with a stronger trend observed in the Pre-DM population, and HbA1c and FPG had a certain contribution to this association. This suggests that LDL-C/HDL-C may serve as an exploratory biomarker for assessing the risk of atherosclerosis in the context of “lipid-glucose comorbidity.”

Carotid atherosclerosis partly reflects cerebral atherosclerosis severity and ranks as a key IS risk factor (18, 19). Carotid plaques offer a window into systemic atherosclerotic disease. For instance, M. Hollander et al. reported a dose-dependent relationship between carotid plaques and IS, stating that carotid plaques in even asymptomatic patients may serve as a source of thromboembolism (20, 21). Additionally, vulnerable carotid plaques have been significantly associated with recurrent stroke (22).

Extensive research shows that lipid profiles play a pivotal role in the formation and progression of carotid atherosclerotic plaques, with dyslipidemia closely linked to IS outcomes (23, 24). Therefore, regular lipid monitoring not only provides a comprehensive view of a patient’s lipid metabolism but also offers an effective foundation for preventing and treating cerebrovascular diseases. This study confirms that the LDL-C/HDL-C ratio, a novel lipid biomarker, dynamically reflects the combined effects of pro-atherosclerotic and anti-atherosclerotic processes. It outperforms traditional single lipid parameters as a risk predictor for carotid plaques and has certain reference value in the risk identification of IS events. Prior studies indicate that LDL-C positively correlates with atherosclerosis development and progression. Lowering LDL-C levels can effectively reduce atherosclerotic disease incidence, while the duration and severity of elevated LDL-C are closely tied to atherosclerotic cardiovascular disease risk (7, 25). In contrast, HDL exerts protective effects against atherosclerosis (26, 27) by promoting reverse cholesterol transport: it shuttles free cholesterol from peripheral cells to the liver for excretion. This mechanism reduces plaque formation, enhances plaque stability, and lowers overall disease risk (28, 29). Lipoprotein ratios capture interactions among lipid components. The LDL-C/HDL-C ratio specifically represents the balance between anti-atherosclerotic and pro-atherosclerotic lipoproteins, and an imbalance in this ratio elevates cardiovascular risk (30). Integrating LDL-C/HDL-C into risk assessment systems thus helps identify patients underestimated by conventional markers and provides a basis for risk stratification in patients with IS.

This study’s most significant finding is that the association between LDL-C/HDL-C and carotid plaque risk was more prominent in the Pre-DM population. This relationship likely stems from IR. Pre-DM marks a critical phase where IR is active but β-cell function remains compensated (31, 32). IR drives dyslipidemia, including reduced plasma HDL, emergence of sdLDL, and elevated TG (10, 33, 34). Reportedly, IR leads to abnormal LDL particle composition, promoting faster vascular wall penetration, greater oxidation susceptibility, and enhanced macrophage uptake, thereby promoting atherosclerosis (35–37). Additionally, HDL particles in IR show reduced phospholipids and apolipoprotein E content, further contributing to atherosclerosis (38–40). The LDL-C/HDL-C ratio may precisely capture the early synergistic interplay between “lipid metabolism disorder” and “glucose metabolism abnormality” during this phase. This synergy of LDL-C/HDL-C is absent in individuals with NGR and attenuated in patients with DM due to complex pathophysiological mechanisms involving advanced vascular lesions and complications. Thus, this study suggests that the association between LDL-C/HDL-C and carotid plaque was stronger in the Pre-DM stage. This finding may serve as a signal of “lipid-glucose comorbidity” synergistically accelerating atherosclerosis, suggesting that focusing on LDL-C/HDL-C in the early stage of abnormal glucose metabolism has certain clinical significance.

Exploratory mediation analysis showed that HbA1c and FPG explained 11.5% and 10.5% of the association, respectively. This finding is consistent with the pathophysiological context of IR, which is not only a core pathological link in hyperglycemia but also induces characteristic atherogenic dyslipidemia (10). Subgroup analysis confirmed the positive LDL-C/HDL-C–carotid plaque correlation across sexes, age groups, smoking and drinking statuses, and histories of hypertension. This finding suggests that this indicator has broad applicability and can assist traditional indicators in improving the risk assessment system.

## 5 Strengths and limitations

The strength of this study lies in the fact that, as a single-center, large-sample retrospective study, it effectively ensured data accuracy and internal consistency. The sufficient sample size and strict inclusion and exclusion criteria minimized potential bias. Unlike previous studies that were largely based on the general population or patients with coronary heart disease, we focused on a secondary prevention population with a high burden of metabolic disorders—specifically, patients with first-episode ischemic stroke—thereby providing quantitative evidence for lipid risk assessment in this patient population. Furthermore, we further focused on the “gray zone” of prediabetes and found that this stage may represent a potential window for initiating lipid risk assessment. Early attention to LDL-C/HDL-C levels may have certain value for IS risk assessment and secondary prevention.

This study also has several limitations. First, as a cross-sectional study, data were collected at a single time point, which precludes establishing the temporal sequence among exposure, mediators, and outcomes, as well as causal inference. Future prospective studies are needed for further validation. Second, the study population consisted exclusively of patients with IS, and the prevalence of carotid artery plaque was relatively high, which limits the generalizability of the findings to some extent. Third, to explore the independent association between LDL-C/HDL-C and carotid plaque risk under natural conditions, this study excluded individuals receiving lipid-lowering drug interventions, which may limit the applicability of the conclusions to populations on lipid-lowering medications. Finally, the predictive performance of LDL-C/HDL-C may vary across different stroke subtypes. Future studies should collect complete data on stroke etiological subtypes (TOAST classification) and conduct further analyses within a prospective design to more comprehensively evaluate its clinical utility.

## 6 Conclusion

This study demonstrates a significant positive association between LDL-C/HDL-C levels and carotid plaque risk in patients with first-ever IS, with a higher association strength particularly observed in Pre-DM cohort. These results highlight that clinicians may consider LDL-C/HDL-C levels as an important reference indicator for assessing the synergistic harm of “lipid-glucose comorbidity,” guiding risk screening in high-risk populations to achieve secondary prevention of IS events.

## Data Availability

The raw data supporting the conclusions of this article will be made available by the authors, without undue reservation.
