# Supplementary material for: Investigating the association between low-density lipoprotein cholesterol/high-density lipoprotein cholesterol and the risk of carotid artery plaque in patients with first-ever ischemic stroke based on different glucose metabolic conditions
Source: Front Endocrinol (Lausanne). 2026 Jul 9;17:1883414. doi: 10.3389/fendo.2026.1883414 (PMC13391398; doi:10.3389/fendo.2026.1883414)
Supplement: Supplementary file 1 [file DataSheet1.docx]

**Table S1** Baseline characteristics of included and excluded participants

| **Characteristics** | **Included participants（N=12166）** | **Excluded participants（N=48862）** | **P-value** |
| --- | --- | --- | --- |
| **Sex** |  |  | **0.68** |
| Male，n（%） | 7805（64.2） | 31250（64.0） |  |
| Female，n（%） | 4361（35.8） | 17612（36.0） |  |
| **Age，year** | 64.0（58.0-72.0） | 64.0（58.0-72.0） | **0.85** |
| **Smoking，n（%）** | 5967（49.0） | 23856（48.8） | **0.82** |
| **Drinking，n（%）** | 4850（39.9） | 19487（39.9） | **0.96** |
| **History of hypertension，n（%）** | 8151（67.0） | 32764（67.1） | **0.74** |
| **History of diabetes，n（%）** | 4965（40.8） | 19895（40.7） | **0.99** |
| **NIHSS** | 4.0（2.0-7.0） | 4.0（2.0-7.0） | **0.72** |
| **HbA1c, %** | 6.2（5.6-7.4） | 6.2（5.6-7.4） | **0.88** |
| **FPG，mmol/L** | 5.7（4.9-7.3） | 5.7（4.9-7.3） | **0.65** |
| **TC，mmol/L** | 4.1（3.4-4.9） | 4.1（3.4-4.9） | **0.91** |
| **TG，mmol/L** | 1.3（1.0-1.8） | 1.3（1.0-1.8） | **0.78** |
| **HDL-C，mmol/** | 1.0（0.9-1.2） | 1.0（0.9-1.2） | **0.84** |
| **LDL-C，mmol/L** | 2.4（1.8-3.1） | 2.4（1.8-3.1） | **0.93** |
| **Carotid Plaque，n（%）** | 9469（77.8） | 37750（77.3） | **0.18** |

Abbreviations: NIHSS, National Institutes of Health Stroke Scale; HbA1c, glycosylated hemoglobin; FPG, fasting plasma glucose; TC, total cholesterol; TG, triglycerides; HDL-C, high-density lipoprotein cholesterol; LDL-C, low-density lipoprotein cholesterol.

**Table S2** Incremental Predict Value of LDL-C/HDL-C for Carotid Plaque Risk: Reclassification Analysis

| **Model** | **X^2^(df)** | **P-value** | **NRI** | **P-value** | **IDI** | **P-value** |
| --- | --- | --- | --- | --- | --- | --- |
| **Clinical Risk Factors** | 1113.3 (8) | <0.001 | Reference | Reference | Reference | Reference |
| **Clinical Risk Factors**  **+LCL-C/HDL-C** | 1144.1 (9) | <0.001 | 0.015  (0.003-0.028) | 0.007 | 0.0026  (0.0016-0.0037) | <0.001 |

NRI: the net reclassification improvement; IDI: integrated discrimination improvement;

model: adjusted for sex, age, smoking status, drinking status, hypertension, NIHSS, TC, TG.


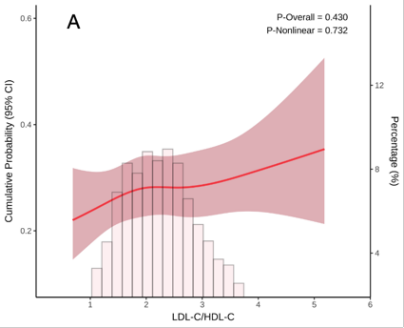

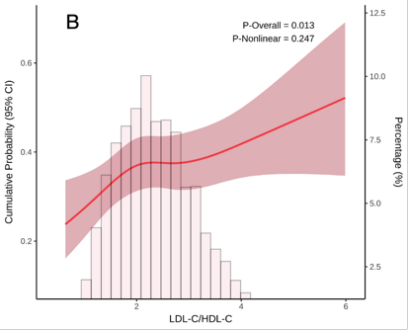

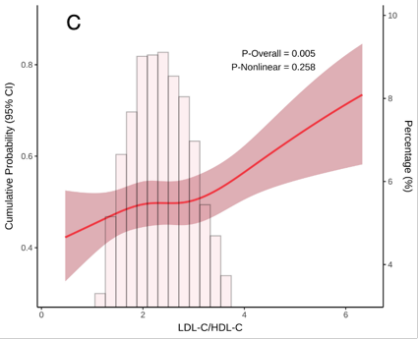


A, participants with NGR; B, participants with Pre-DM. C, participants with DM. The dark red line represents the reference for the odds ratio (OR), the light red area represents the 95% confidence interval (CI), and the histogram represents the distribution of LDL-C/HDL-C. The model was adjusted for sex, age, smoking status, drinking status, hypertension, NIHSS, TC, and TG.

**Fig. S1** Restricted cubic spline curve for the association between LDL-C/HDL-C and the risk of carotid plaques according to different glucose regulation states

**Table S3** Exploratory Mediating Effects in the Association Between LDL-C/HDL-C and the Risk of Carotid Plaques

| **Exploratory Mediating effects** | **Risk of Carotid Plaques** | | |
| --- | --- | --- | --- |
|  | **Estimate** | **95 % CI** | **P-value** |
| **HbA1c** |  |  |  |
| Total Effect | 0.0286 | （0.0180-0.0400） | <0.001 |
| ADE（average） | 0.0253 | （0.0150-0.0370） | <0.001 |
| ACME（average） | 0.0033 | （0.0019-0.0047） | <0.001 |
| PropMediated（average） | 0.1153 | （0.0626-0.1932） | <0.001 |
| **FPG** |  |  |  |
| Total Effect | 0.0286 | （0.0180-0.0400） | <0.001 |
| ADE（average） | 0.0256 | （0.0151-0.0370） | <0.001 |
| ACME（average） | 0.0030 | （0.0018-0.0044） | <0.001 |
| PropMediated（average） | 0.1049 | （0.0574-0.1781） | <0.001 |

95%CI, 95% Confidence interval.

**Table S4** Sensitivity analysis for the association between LDL-C/HDL-C and carotid plaque risk

| **Characteristics** | **PR (95% CI)** | **P-value** |
| --- | --- | --- |
| LDL-C/HDL-C | 1.033 (1.021–1.045) | <0.001 |

95%CI, 95% Confidence interval; PR: the prevalence ratio estimated by Poisson regression with robust variance; adjusted for sex, age, smoking status, drinking status, hypertension, NIHSS, TC, TG.

**Table S5** Sensitivity analysis: E-values for the association between LDL-C/HDL-C and carotid plaque risk

| **Characteristics** | **OR (95% CI)** | **E-value (point)** | **E-value (CI limit)** |
| --- | --- | --- | --- |
| LDL-C/HDL-C | 1.224 (1.147–1.305) | 1.26 | 1.21 |

E-value, calculated to assess the minimum strength of association that an unmeasured confounder would need to have with both the exposure and the outcome to fully explain the observed association. The E-value for the confidence interval limit is based on the lower bound of the 95% CI;

adjusted for sex, age, smoking status, drinking status, hypertension, NIHSS, TC, TG.
